# Benzodiazepine use in hospitalized older adults: a retrospective observational study

**DOI:** 10.1097/j.pbj.0000000000000303

**Published:** 2025-10-01

**Authors:** Sandra Torres, Marta Martins, Gonçalo Sarmento

**Affiliations:** a São João Family Health Unit (São João da Madeira), Unidade Local de Saúde Entre Douro e Vouga, São João da Madeira João da Madeira, Portugal; b Acute Geriatric Unit, Department of Internal Medicine, Unidade Local de Saúde Entre Douro e Vouga, Santa Maria da Feira, Portugal

**Keywords:** Benzodiazepines, chronic medication, elderly population, deprescribing, hospitalization

## Abstract

**Background:**

To assess the prevalence and indications for the use of benzodiazepines (BZDs) in patients admitted to an acute geriatric unit and to evaluate changes in their prescriptions.

**Methods:**

BZD indications were investigated using computerized clinical records. Changes in BZD prescriptions were assessed at the time of discharge.

**Results:**

Among the 165 patients included (mean age: 86.7 years, 71.5% women), 60 (36.4%) were taking BZD on admission, 58.3% of which were considered inappropriate. At discharge, BZD discontinuation was observed in 11.7% and dose reduction was initiated in 18.3%.

**Conclusions:**

Most patients using BZD had no clear medical indication. Admission to a geriatric ward resulted in successful discontinuation or dose reduction in a third of patients.

## Introduction

Benzodiazepines (BZDs) are drugs that are widely used in the elderly population^1^ and are often prescribed for much longer periods than recommended. The prevalence of long-term BZD use in the elderly varies between 10% and 42%, and there is a positive correlation between age and consumption, with inappropriate prescriptions occurring in two thirds of cases^2^. BZDs are usually indicated for the treatment of generalized anxiety disorder, for a maximum of eight weeks, and insomnia, for a maximum of four weeks, and as adjunctive therapy in schizophrenia and depression^3^. Prolonged use of BZD is associated with the development of dependence, tolerance, and adverse reactions, namely, short-term cognitive impairment (such as memory deficit, learning difficulties, and attention deficit), dementia, increased reaction time, balance and gait disorders, falls, and, consequently, fractures^3-6^. In addition, BZD intake has been shown to increase mortality (1.2–3.7 times higher than in those who do not take BZD)^7^.

The process of deprescribing BZD, dose reduction or discontinuation, can be complex, with healthcare professionals often showing reluctance, fear or lack of confidence in it^8^. Hospitalization in an acute geriatric unit can represent an opportunity to identify and initiate the deprescribing of inappropriately prescribed drugs^9^. The literature on deprescribing during hospitalization is limited, and the subsequent results after hospital discharge remain poorly understood^9^.

Our aim was therefore to assess the chronic use of BZD and deprescribing BZD in elderly people admitted to a geriatric unit, as well as to investigate what happened 3 months after discharge.

## Methods

This retrospective study was carried out in the Acute Geriatrics Unit (AGU) at Hospital São Miguel, part of the Unidade Local de Saúde Entre Douro e Vouga.

All patients admitted in the first 3 months after the unit was set up (from June to August 2024) were included. Patients who died during hospitalization and those readmitted in those 3 months were excluded from the study.

All the information was obtained from computerized clinical records (SClinic and MedTrix).

We compared patients who were taking BZD chronically (more than 3 months) with those who were not. We collected sociodemographic data, functional status (Barthel Index and Modified Rankin Scale), previous history of insomnia, depression, anxiety or other indication for BZD use (including epilepsy), history of falls in the last year, and percentage of patients medicated with more than 5 drugs (polypharmacy).

The statistical analysis used Pearson's chi-square test for gender, type of residence, falls in the last year, and polypharmacy; Student *t* test for age, number of days in hospital, Barthel Index, and Modified Rankin Scale. Prior to applying Student *t* test, the assumption of data normality was verified using appropriate statistical methods, ensuring the validity of the procedure. Statistical significance was set at *P* < .05.

This study was approved by the Ethics Committee of Unidade Local de Saúde de Entre Douro e Vouga (Ethics Committee Registration No. 21_2025). The retrospective nature of the analysis supported the informed consent waiver, for the sake of feasibility. This research ensured the privacy of patient data, since any sort of personal information that allows identification was not used or shown in the database built.

## Results

This study included 165 patients, with an average age of 86.7 ± 5.5 years, 71.5% (N = 118) female and an average length of stay of 9.0 ± 7.6 days. The patients' characteristics are described in Table 1.

**Table 1:** Sociodemographic and clinical characteristics of patients according to benzodiazepine use

	On BDZ (N = 60)	Without BZD (N = 105)	*P-*value	Test statistic
Gender (% women)	71.7	71.4	.974	χ^2^(1) = 0.001
Age (years)	86.36 ± 5.01	86.83 ± 5.79	.624	t(139) = -0.491
Residence			.001*	χ^2^(2) = 13.42
Nursing home (%)	48.3	21.0		
Home (%)	48.3	73.3		
Community Care Unit (%)	3.3	5.7		
Hospitalization days (average)	8.91 ± 5.20	9.66 ± 9.27	.589	t(139)=-0.542
Modified Rankin Scale† (average)	4.30 ± 1.05	4.31 ± 0.81	.975	t(139) = -0.031
Barthel Index‡ (average)	17.83 ± 22.91	20.50 ± 25.93	.538	t(139) = -0.618
Falls over the last year (%)	28.3	25.7	.714	χ^2^(1) = 0.134
Polypharmacy (%)	95.0	81.9	.017*	χ^2^(1) = 5.666

The data has been represented as N, number, and %, percentage. Pearson's chi-square test (chi-square value, X^2^) to calculate *P*-values for gender, type of residence, falls in the last year, and polypharmacy. Student *t* test (t-value, t) to calculate *P*-values for age, number of days in hospital, Barthel Index, and Modified Rankin Scale.

* Indicates statistical significance, representing a *P*-value less than or equal to a predetermined significance level (0.05 or 5%).

† Thirteen patients had no information on the Modified Rankin Scale in their clinical records.

‡ Twenty patients had no information on the Barthel Index in their clinical records.

Before admission, 60 patients (36.4%) were taking chronic BZD, 90% (N = 54) of them daily, and all of them at bedtime. Of the patients taking BZD, 58.3% (N = 35) did not have a clear medical indication suggested in the clinical records for their use. Intermediate half-life BZDs were the most frequently prescribed (N = 31, 51.7%), especially lorazepam (N = 14, 45%). The characteristics of BZD prescriptions are described in Table 2.

**Table 2:** Characteristics of benzodiazepine prescriptions at admission

	On BDZ (N = 60)
Duration of action of BZD	
Short (%)	16 (26.7)
Intermediate (%)	31 (51.7)
Long (%)	13 (21.7)
Indication	
Depression (%)	12 (20.0)
Anxiety (%)	3 (5.0)
Insomnia (%)	2 (3.0)
Other (%)	8 (13.3)
Frequency	
Daily (%)	54 (90.0)
On demand (%)	6 (10.0)
Prescription pattern	
During the day (%)	0 (0.0)
At bedtime (%)	60 (100.0)

The data has been represented as N, number, and %, percentage.

Chronic BZD use was significantly associated with polypharmacy (*P* = .017) and living at nursing home (*P* = .001). There were no significant differences between groups in the percentage of falls in the last year.

**Figure 1.**
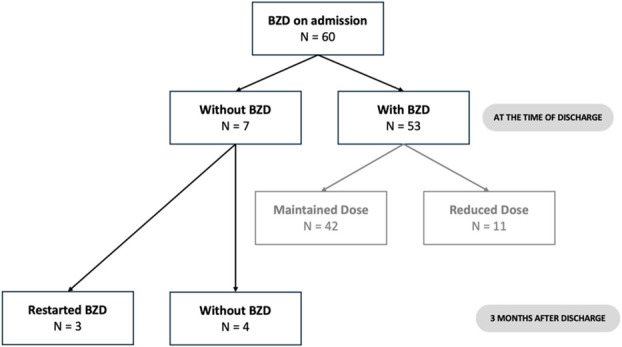
Benzodiazepine prescription status on admission, at discharge, and 3 months after hospitalization. The data has been represented as N, number.

Of the 60 patients on BZD, at the time of discharge, the BZD was replaced by the one with a longer half-life or the dose was reduced in 18.3% (N = 11) and it was suspended in 11.7% (N = 7) of patients (Fig. 1).

Three months after discharge, 3 patients (42.8%) whose BZD had been suspended at the time of discharge, restarted their prescription (by their family doctor or after a visit to the emergency department), and the reintroduction was explained in only one patient (Fig. 1).

## Discussion

Around a third of the elderly admitted to this AGU are chronically medicated with BZD, mainly intermediate half-life BZD. Most of these patients had no clear medical indication for this medication, which is in line with the results of several European studies^1,2,9^.

In this study, chronic BZD use was significantly associated with polypharmacy, which is as expected. This is particularly worrying given that the association of BZD with other drugs that act on the central nervous system and with polypharmacy in general, is associated with an increased risk of falls and fractures and, consequently, high morbidity and mortality^10^.

In addition, of the elderly living at nursing home, there is a significantly higher percentage taking BZD chronically, while of the elderly living at their own homes, there is a significantly higher percentage without a BZD prescription. This may be due to the more individualized care provided at home.

According to the literature, taking BZD is associated with a higher risk of falls^10^. This was not the case in this study. This may be because only falls that took the patient to the emergency department were counted.

The deprescription rate described in the literature is around 40%^10^. During the first 3 months of this AGU existence, the deprescription rate was 30%. However, this deprescribing is not difficult to maintain in an outpatient setting for 3 months, where the indication to resume taking BZD is limited.

Therefore, the hospitalization of these patients was an important opportunity to suspend or reduce the dose of BZD prescribed. Despite this, the process of deprescribing is complex and more training and guidance is needed for health professionals to integrate and promote it in their usual clinical practice. In addition, the provision of educational material and continuous monitoring of these elderly people seems to achieve a gradual reduction in the chronic use of BZD in this population^3,10^. Melatonin replacement, with or without psychological support, has also been shown to contribute to the success of BZD deprescription and could be another technique to implement^11^.

The importance of this study lies in its ability to describe the prevalence of BZD use and its management in an AGU. The number of patients included, as well as the average age, is like previous studies. In addition, it provides an opportunity to show the importance of the existence of this AGU and the benefits of its creation, particularly regarding specialized care dedicated to the elderly population.

However, this study has several limitations, including its retrospective nature and the fact that it is a single center, so data collection and follow-up of these patients was restricted. It was not possible to assess the initial indication for prescribing BZD. In addition, the lack of data on the concomitant use of drugs that act on the central nervous system or others, which can also promote the risk of falls and other complications, was also a limitation. Another limitation was the lack of follow-up of these patients after deprescribing, to assess withdrawal symptoms, sleep patterns, and impact on quality of life, which would have improved understanding of this intervention.

To maintain the process of deprescribing and discontinuing BZD in the long term, a multifactorial approach is essential. More research is needed to assess the impact of specific interventions on deprescribing in various contexts, particularly during hospitalization, where the existing literature is limited.

In conclusion, although hospitalization in an AGU provides an opportunity for BZD deprescribing to be promoted, it is also crucial to consider the post-discharge stage and the implementation of general recommendations to ensure the continued success of this important clinical practice.
